# Numerical investigation of cavitation in periodontal Pockets: Insights for enhancing cleaning efficiency

**DOI:** 10.1016/j.ultsonch.2023.106625

**Published:** 2023-09-30

**Authors:** You Yu, Warren R. Smith, Qianxi Wang, A. Damien Walmsley

**Affiliations:** a School of Mathematics, University of Birmingham, Birmingham B15 2TT, UK; b School of Dentistry, College of Medical and Dental Sciences, University of Birmingham, Birmingham B5 7SA, UK

## Abstract

Ultrasonic dental scalers are indispensable instruments for efficient dental cleaning through the generation of cavitation. To gain valuable insights and enhance the cavitation cleaning effects, a numerical investigation is conducted using the finite element method via ABAQUS. Numerical results are compared with the experimental cavitation image for a scaler undergoes vibrations near a wall. We then analyse how the amplitude, frequency, and cross-sectional shape of the scaler affect cavitation generation. Numerical results indicate that cavitation is more pronounced for a scaler oscillating near a nearly rigid boundary than a soft boundary. It increases with the vibration amplitude because of higher ultrasonic energy transferring to the liquid and generating stronger pressure waves. The resonant frequency of the scaler coincides with the maximum cavitation and scaler tip amplitude. Reducing the dimension of the cross-section of the scaler in its oscillation direction increases both the scaler tip amplitude and the cavitation generated. This finding offers a potential design approach for enhancing the scaler cavitation and its cleaning effects. These insights provide practical guidance for optimising dental scaler settings, which can improve oral hygiene and prevent complications related to dental implants.

## 1 Introduction

Ultrasonic dental scalers are essential instruments in routine periodontal therapy for effectively removing a bacterial biofilm (also known as dental plaque) [1]. The optimal treatment for cleaning dental implant surfaces effectively has not been established although the use of ultrasonic instruments is recommended [2].

Cavitation includes the creation and subsequent collapse of microbubbles within a liquid, resulting in the generation of microjets, shockwaves, and micro-streamers that contribute to the surface cleaning effects [3], [4], [5], [6]. Previous research has confirmed the significant role of cavitation generated by ultrasonic dental scalers in dental implants cleaning [4], [7], [8], [9]. Manmi et al. [10] explored the effects of operating conditions of ultrasound scalers, such as amplitude and frequency, on cavitation generation around an ultrasonic dental scaler within a liquid without any bound. Yu et al. [11] investigated cavitation generation by a dental scaler within a simplified periodontal pocket with a rigid boundary.

This paper describes two developments to the above research. Firstly, we will account for the influence of soft tissue of the periodontal pocket to cavitation development, which better represents the practical clinical scenario. Secondly, we will study the effects of the scaler's cross-sectional shape on cavitation generation to optimize the design of ultrasonic scalers.

Researchers have extensively studied the mechanical characteristics of soft tissue, resulting in constitutive models grounded in the hyperelasticity theory [12], [13], [14] that precisely capture the nonlinear behaviour of soft tissue. A frequently employed model for soft tissue is the hyperelastic Ogden model [15], [16]. We incorporate the Ogden model to characterise the hyperelastic deformation of gum tissue. An isotropic linear elastic theory which is commonly applied to model stainless steel [17] is used for modelling the ultrasonic dental scaler. The linear potential flow theory is used for modelling the liquid part. The kinetic and dynamic boundary conditions are incorporated to account for interactions between the scaler tip, liquid, and gum tissue.

Cavitation phenomena are generally associated with bubble clusters, but a comprehensive understanding of the intricate dynamics of these clusters remains elusive. In practical engineering applications, cavitation bubble clusters are often modelled using simplified models [18], [19], [20], [21], [22], [23], [24], [25], [26], [27] that neglect bubble–bubble interactions and the resulting non-spherical deformation of bubbles. Hydrodynamic cavitation is a result of gas evaporation under pressures below the vapour pressure [28], [29]. In our research, we employ a pressure cut-off cavitation model, which predicts cavitation zones based on pressure falling below the saturated vapour pressure. This model has the capability to accurately estimate global cavitation zones [26], [27].

The physical and numerical model will be described in section 2. Validation will be conducted in section 3, comparing the numerical results with experimental cavitation images for a scaler vibrating near a wall. Parametric studies investigating the effects of vibration amplitude, frequency, and cross-sectional shapes of the dental scaler on cavitation generation will be presented in section 4. Finally, section 5 will provide conclusions and insights to enhance the cleaning efficiency of dental scalers in a non-contact mode.

## 2 Physical and numerical model

### 2.1 Problem description

**Fig. 1:**
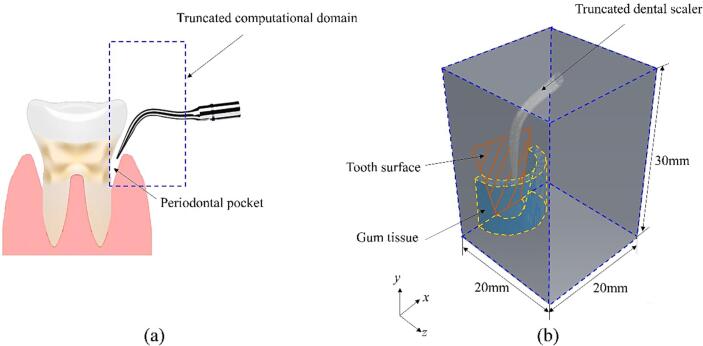
(a) Schematic illustration of the ultrasonic dental scaler and the periodontal pocket, (b) 3D truncated domain in Cartesian coordinates.

**Fig. 2:**
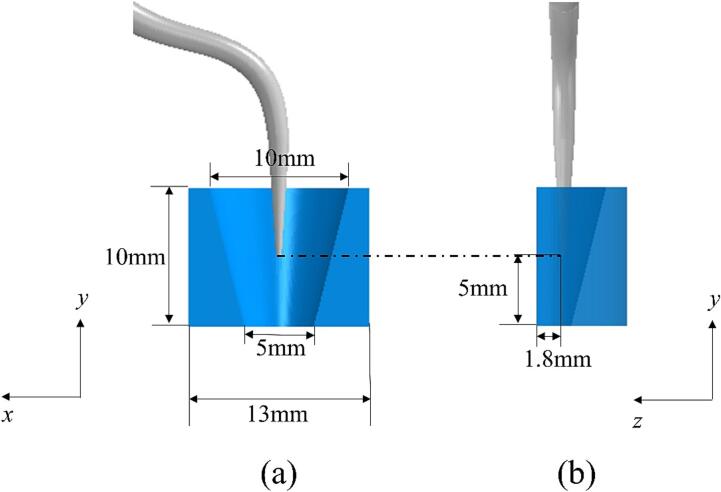
The simplified representation of the periodontal pocket with gum tissue and the positions of the scaler in (a) the *x-y* plane, and (b) the *y-z* plane.

An ultrasonic dental scaler is assumed to undergo vibrations within a water-filled periodontal pocket, as illustrated in Fig. 1a. A cubic truncated computational domain with dimensions 20 mm × 30 mm × 20 mm in the *x*, *y*, and *z* directions is selected, as depicted in Fig. 1b. The tooth surface is represented as a rigid plane, and the periodontal pocket with gum tissue is modelled as a circular cylinder with a radius of 6.5 mm having a truncated cone removed with the cone radius changing from 5 mm to 2.5 mm and a cone depth of 10 mm, as depicted in Fig. 2. The scaler tip is located 5 mm deep in the pocket and vibrates parallel to the tooth surface plane along the *x*-axis, maintaining a 1.8 mm distance from the tooth surface.

**Fig. 3:**
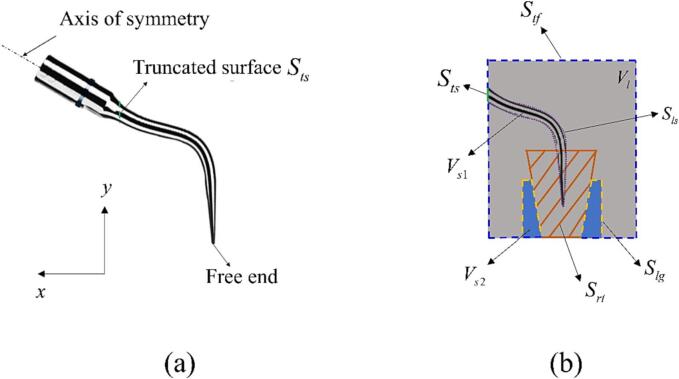
(a) The ultrasonic dental scaler used in the simulations. (b) Cross-sectional view of the computational domain at the *x-y* plane.

Previous studies [10], [11] have shown that the scaler tip displacement remains unaffected by the truncated surface when the truncation is near the circular cylinder base. Consequently, in this study, the scaler is truncated at *S_ts_*, as depicted in Fig. 3a, to reduce the computational domain size and save CPU time. We define a harmonic oscillation at the truncated surface *S_ts_* of the scaler with the direction along the axis of symmetry in Fig. 3a and the displacement of *A*sin(2π*ft*), where *A* denotes the amplitude and *f* represents the frequency. As shown in Fig. 3b, the scaler is denoted as *V_s_*_1_ and its surface *S_s_* = *S_ls_* ∪ *S_ts_*, where *S_ls_* represents the liquid-scaler interface. The gum tissue is denoted as *V_s_*_2_, and the liquid-tissue interface is denoted as *S_lg_*. The truncated liquid domain is represented by *V_l_*, bounded by the scaler surface *S_ls_*, the gum tissue surface *S_lg_*, the tooth surface *S_rt_*, and all the remaining truncated planes *S_tf_*.

### 2.2 Structure modelling

In this work, the structure, including both the scaler and the gum tissue, is governed by the momentum equation [31]

(1) $$\rho_{\mathrm{str}}{\ddot{u}}_{\mathrm{str}}-\nabla \cdot \sigma_{\mathrm{str}}=\rho_{\mathrm{str}}b,$$

where *ρ_str_* represents the density of the structure, ${\ddot{u}}_{\mathrm{str}}$ denotes the acceleration of a material particle of the structure, and the body forces acting on the structure, including damping and gravity, are denoted by **b**. We calculate the Cauchy stress tensor **σ***_str_* for the structure by applying Hooke's law to the linear elastic material of the scaler and the “rigid” periodontal pocket, while utilizing the Ogden model for the hyperelastic material of the “soft” periodontal pocket.

The Ogden model [15], [16] has the following the strain energy function

(2) $$U\left(\lambda_{1},\lambda_{2},\lambda_{3}\right)=\sum_{i=1}^{n}\frac{\mu_{i}}{\alpha_{i}}\left(\lambda_{1}^{\alpha_{i}}+\lambda_{2}^{\alpha_{i}}+\lambda_{3}^{\alpha_{i}}-3\right),$$

where *λ_j_*, *j* = 1, 2, 3, is the principal stretches, *μ_i_* and *α_i_* are material parameters of the Ogden model, which can be determined from material tests, and *n* is the order of the Ogden model and *n* = 3 is adopted in this paper. The Cauchy stress **σ***_str_* is obtained by differentiating the strain energy density function *U*(*λ*) with respect to the stretch *λ* [15], [16].

As mentioned in Yu et al. [11], an equivalent weak form for finite element analysis when neglecting all body forces **b** can be expressed as

(3) $$\int_{V_{s}}\delta \varepsilon_{\mathrm{str}}:\sigma_{\mathrm{str}}dV+\int_{V_{s}}\rho {\;}_{\mathrm{str}}\delta u_{\mathrm{str}}\cdot {\ddot{u}}_{\mathrm{str}}dV+\int_{S_{\mathrm{ls}}\cup S_{\mathrm{lg}}}p_{\mathrm{str}}\delta u_{\mathrm{str}}\cdot ndS-\int_{S_{\mathrm{ts}}}\delta u_{\mathrm{str}}\cdot t_{\mathrm{str}}dS=0,$$

where *δ***u***_str_* is an arbitrary variation displacement field, *δ***ε***_str_* is the corresponding strain variation, **n** denotes the outward unit normal to the surface of the structure, *p_str_* represents the pressure exerted on both the liquid-scaler interface *S_ls_* and the liquid-tissue interface *S_lg_*, and **t***_str_* represents the surface traction which is calculated from the harmonic oscillation at *S_ts_*.

### 2.3 Liquid modelling

Following the linear potential flow theory, an inviscid, compressible, and irrotational liquid is governed by [31], [32], [33], [34]

(4) $$\nabla p_{\mathrm{liq}}+\gamma {\dot{u}}_{\mathrm{liq}}+\rho_{\mathrm{liq}}{\ddot{u}}_{\mathrm{liq}}=0,$$

where *ρ_liq_* is the liquid density, *p_liq_* is the pressure in the liquid that satisfies the linear constitutive behaviour $p_{\mathrm{liq}}=-K_{\mathrm{liq}}\nabla \cdot u_{\mathrm{liq}}$, with *K_liq_* being the bulk modulus of the liquid and **u***_liq_* being the displacement of the liquid particle. The dot and double dot denote the first and second order time derivatives, and volumetric drag coefficient *γ* is disregarded in our simulations due to its minor effect in comparison to the liquid's inertial forces.

The equivalent weak form of the momentum equation for finite element analysis of the liquid can be written as

(5) $$\int_{V_{f}}\left(\frac{1}{K_{\mathrm{liq}}}{\ddot{p}}_{\mathrm{liq}}\delta p_{\mathrm{liq}}+\frac{1}{\rho_{\mathrm{liq}}}\nabla \delta p_{\mathrm{liq}}\cdot \nabla p_{\mathrm{liq}}\right)dV+\int_{S_{\mathrm{rt}}\cup S_{\mathrm{ls}}\cup S_{\mathrm{lg}}\cup S_{\mathrm{tf}}}T_{\mathrm{liq}}\left(x\right)\delta p_{\mathrm{liq}}dS=0,$$

(6) $$T_{\mathrm{liq}}\left(x\right)=-\frac{n\cdot \nabla p_{\mathrm{liq}}}{\rho {\;}_{\mathrm{liq}}}=n\cdot {\ddot{u}}_{\mathrm{liq}},$$

where $\delta p_{\mathrm{liq}}$ is an arbitrary variation pressure field, ***x*** is the spatial position, and *T_liq_*(***x***) is the boundary traction, determined by the boundary conditions on surfaces *S_rt_*, *S_ls_*, *S_lg_* and *S_tf_*. For the tooth surface *S_rt_*, a rigid immobile wall is applied, while for the truncated surfaces *S_tf_* of the liquid domain, a non-reflective boundary condition is implemented to avoid the reflection of pressure waves from the truncated boundaries. The kinetic and dynamic boundary conditions at the interfaces *S_ls_* and *S_lg_* are employed to model the interactions between the liquid-scaler and liquid-tissue, respectively. Further details of these boundary conditions and the derivation of equations (3), (5) can be found in Yu et al. [11].

### 2.4 Cavitation modelling

Hydrodynamic cavitation occurs in the liquid when the absolute pressure drops below the saturated vapour pressure of the liquid *p_c_*. In the pressure cut-off cavitation model in ABAQUS [31], the pressure *p_liq_* within the liquid is then determined as

(7) $$p_{\mathrm{liq}}=\text{max}\left\{p_{\mathrm{liq}},p_{c}\right\}.$$

This allows us to capture the instantaneous effects of cavitation and incorporate them into the overall model.

## 3 Numerical validations

### 3.1 Numerical setup

**Fig. 4:**
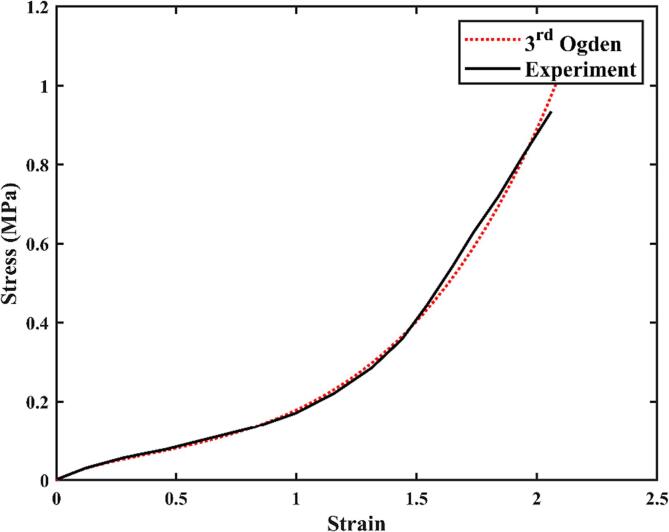
Strain-stress curves of PDMS obtained from the experiment [30] and the fitting results using the 3rd Ogden model in the simulations.

Parameters used for liquid modelling include: the liquid density *ρ_liq_* = 1000 kg/m^3^, the liquid bulk modulus *K_liq_* = 2140 MPa, and the saturated vapour pressure of the liquid *p_c_* = 2300 Pa. The scalers and the periodontal pocket with a rigid boundary (also referred to as “rigid pocket”) are modelled using the following parameters: the density *ρ_str_*_1_ = 8000 kg/m^3^, the Young's modulus *E* = 224 GPa, and Poisson’s ratio *ν* = 0.3 [35]. Polydimethylsiloxane (PDMS) is a hyperelastic material widespread in biomedical research and technology, often employed as a substitute for soft tissue [36]. The density of PDMS is *ρ_str_*_2_ = 980 kg/m^3^ and its hyperelastic properties [37] are represented by the 3rd order Ogden model in our simulations to mimic soft gum tissue around the periodontal pocket (also referred to as “soft pocket”). Strain-stress curves are presented in Fig. 4, and the material parameters for the 3rd order Ogden model are *μ*_1_ = -0.102 MPa, *μ*_2_ = -0.095 MPa, *μ*_3_ = -0.117 MPa, *α*_1_ = 5.226, *α*_2_ = 5.448, and *α*_3_ = -5.545.

Varying densities are adopted for the discretisation of the computational domain. The scaler component is modelled using approximately 0.1 million C3D4 solid elements, which are commonly employed for modelling solid structures. The mesh sizes exhibit a range, ranging from 0.01 mm in proximity to the scaler tip where greater displacements are anticipated, to 0.06 mm closer to the truncated surface of the scaler. The fluid domain is meshed with acoustic elements (AC3D4), progressively growing from 0.015 mm near the scaler surface to 0.2 mm at the outer boundary of the fluid domain. The gum tissue is meshed with C3D8 solid elements with uniform size of 0.1 mm. The computational domain contains a total of approximately 8 million elements, in accordance with the aforementioned criteria. ABAQUS will utilize the finite element method (FEM) to discretize and solve the governing equations detailed in section 2 over the mesh elements to simulate the three-dimensional, nonlinear, and transient interactions among scaler vibration, gum tissue deformation, liquid flow, and cavitation.

### 3.2 Mesh convergence test

**Fig. 5:**
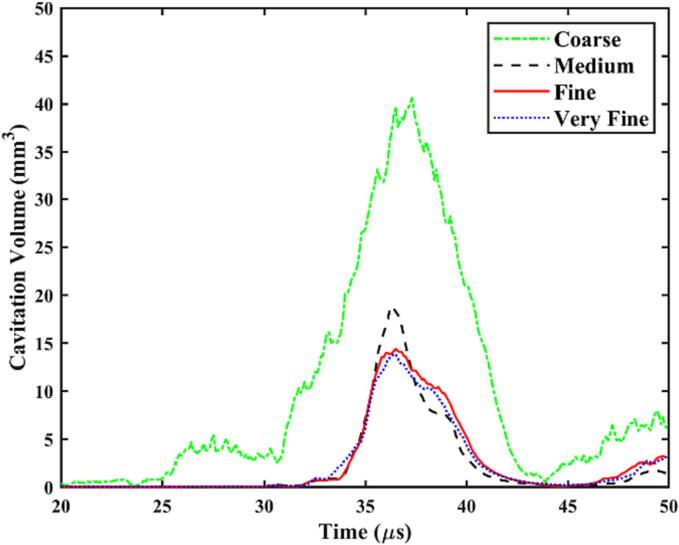
Mesh convergence tests for the cavitation volume within the periodontal pocket over time. The element numbers are about 1 million, 3 million, 8 million, and 12 million. The truncated surface of the scaler oscillates harmonically at the amplitude *A* = 0.01 mm and the frequency *f* = 31 kHz. Other parameters for the calculations are *ρ_liq_* = 1000 kg/m^3^, *K_liq_* = 2140 MPa, *p_c_* = 2300 Pa, *ρ_str_*_1_ = 8000 kg/m^3^, *E* = 224 GPa, *ν* = 0.3, *ρ_str_*_2_ = 980 kg/m^3^, *μ*_1_ = -0.102 MPa, *μ*_2_ = -0.095 MPa, *μ*_3_ = -0.117 MPa, *α*_1_ = 5.226, *α*_2_ = 5.448, and *α*_3_ = -5.545.

The convergence test is carried out using varying numbers of elements, including coarse (about 1 million), medium (about 3 million), fine (about 8 million), and very fine (about 12 million). The results, displayed in Fig. 5, show cavitation volume history within the periodontal pocket. The truncated surface of the scaler oscillates harmonically at the amplitude *A* = 0.01 mm and the frequency *f* = 31 kHz. Similar results are obtained for the two sets of mesh with higher resolutions. Consequently, the mesh resolution employed for the fine mesh consisting of 8 million elements, as detailed in section 3.1, is applied to all subsequent simulations.

### 3.3 Comparison with the experimental data

**Fig. 6:**
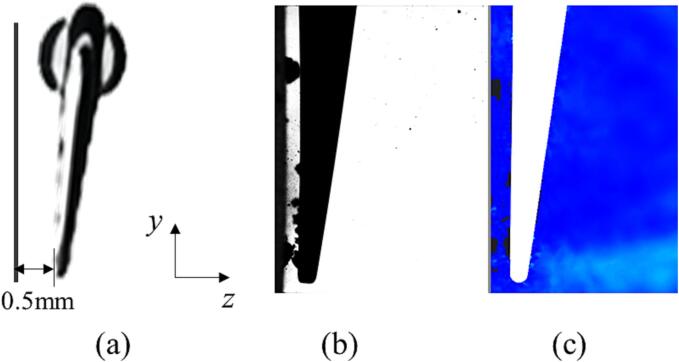
(a) Schematic of the scaler near a wall in the experiment, (b) a high-speed experimental cavitation image, and (c) the contour of the liquid pressure. The black region represents cavitation, where the pressure reduces to the cavitation limit of 2300 Pa. Other parameters are the same as in Fig. 5.

To validate the numerical model, we compare the numerical results of the cavitation pattern with the experimental image when an ultrasonic dental scaler undergoes vibrations in close proximity to a wall. The experimental image was acquired from an ultrasonic dental scaler undergoes vibrations in a water tank. More details of the experiment setup can be found in Vyas et al. [9]. As shown in Fig. 6a, the scaler oscillates parallel to the wall in the experiment, maintaining a 0.5 mm distance from the wall, replicating a clinical setting aimed at avoiding potential damage from accidental contact with the wall. In this specific case, the gum tissue *V_s_*_2_ in Fig. 3b is set as liquid to simulate the scaler's proximity to a wall.

Previous experiments and simulations were conducted in an unbounded domain, revealing cavitation clouds occurring on both sides of the scaler tip in the direction of displacement [9], [10], [11]. However, cavitation occurs on the side of the tip closest to the wall with the proximity to a wall in both experiments and simulations (as shown in Fig. 6b and 6c). The cavitation pattern in the simulation closely matches the experimental results.

## 4 Numerical results

In clinical settings, ultrasonic dental scalers often operate at frequencies ranging from 20 to 51 kHz, and the amplitude of oscillation can be adjusted by varying the input power of the instrument. We conduct parametric studies to investigate the effects of the oscillation amplitude *A* and frequency *f* of the dental scaler on cavitation volume within the periodontal pocket. Additionally, we explore the influence of the cross-sectional shape of the scaler tip on cavitation generation. The simulation is conducted for a duration of 500 *μ*s, and the time-averaged void fraction in the periodontal pocket, representing the amount of cavitation generated and indicating the cleaning effects implicitly, is evaluated.

### 4.1 Effects of the oscillation amplitude on cavitation generation

To analyse the impact of the scaler's oscillation amplitude *A* on cavitation generation, we simulate the dental scaler oscillating in the periodontal pocket with both soft and nearly rigid (by setting the gum tissue with the same material as the scaler) boundaries at various amplitudes *A* ranging from 0.005 to 0.02 mm and a frequency *f* of 31 kHz.

**Fig. 7:**
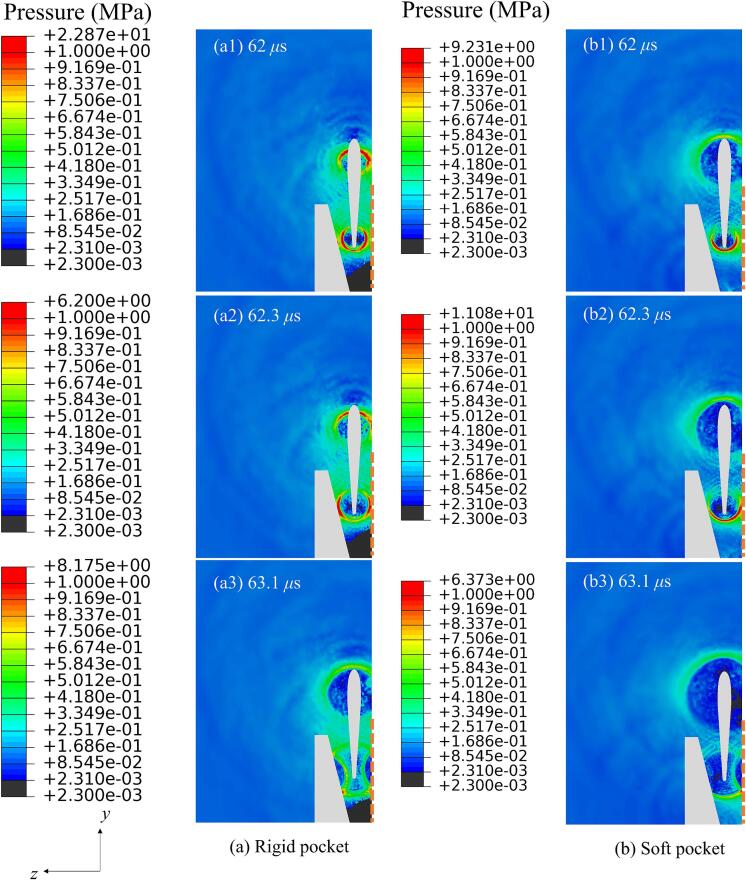
Pressure contours from the *yz*-plane, illustrating the propagation of acoustic pressure waves within periodontal pockets with (a) a rigid boundary and (b) a soft boundary for the cases with *A* = 0.01 mm and *f* = 31 kHz. Other parameters are the same as in Fig. 5. The dashed line on the right side of each frame represents the rigid tooth surface.

Fig. 7 illustrates the propagation of acoustic pressure waves within the periodontal pockets with *A* = 0.01 mm and *f* = 31 kHz. The dashed line on the right side of each frame represents the rigid tooth surface. In the case of a scaler oscillating within a rigid pocket, as shown in Fig. 7a, the acoustic pressure wave propagates with a circular cross-section within the pocket until it encounters the surfaces of the pocket and the tooth. Subsequently, distinct reflections of the pressure waves are observed from both the pocket and the tooth surfaces in frame 3 of Fig. 7a. The behaviour of pressure wave propagation within a soft pocket is similar to that within a rigid pocket, with the exception that the pressure wave significantly weakens upon interaction with the soft pocket surface, as evidenced in frame 3 of Fig. 7b.

**Fig. 8:**
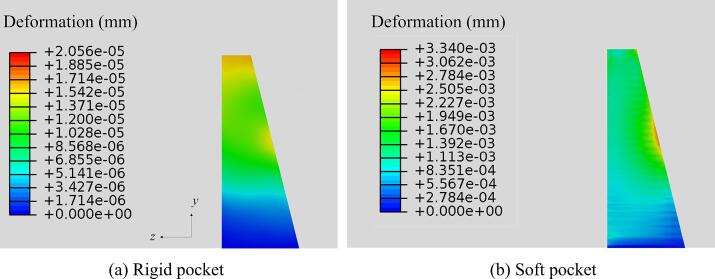
Deformation contours from the *yz*-plane illustrating the deformation of the periodontal pockets with (a) a rigid boundary and (b) a soft boundary for the cases in Fig. 7.

**Fig. 9:**
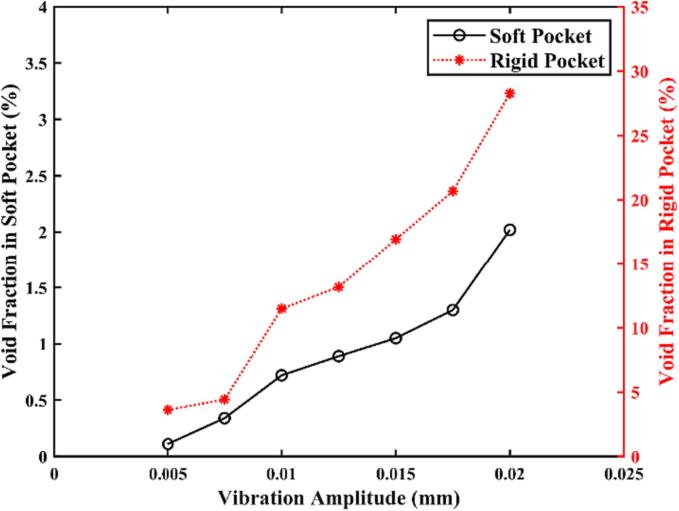
Void fraction in the periodontal pocket with both soft and nearly rigid boundaries plotted against the oscillation amplitude *A* of the scaler tip. The oscillation amplitude *A* ranges from 0.005 to 0.02 mm, and other parameters are the same as in Fig. 5.

Fig. 8 depicts the deformation of both the rigid and soft pockets at 63.1 *μ*s. When the scaler oscillates within a periodontal pocket, the deformation of a soft pocket is approximately two orders of magnitude greater than that of a rigid pocket. This indicates that a significant portion of the ultrasonic energy transmitted into the liquid is absorbed by the soft boundary and converted into elastic wave energy, rather than being reflected at the rigid boundary. Consequently, a substantially lower level of cavitation occurs when the pocket boundary is soft as shown in Fig. 9.

Fig. 9 also illustrates that the void fraction increases with the vibration amplitude for both soft and nearly rigid boundaries. The energy density of a linear plane harmonic acoustic wave can be estimated as

(8) $$I=\frac{1}{2}\rho_{f}c{\left(2\pi f_{p}\right)}^{2}A_{p}^{2},$$

where *f_p_* and *A_p_* are the oscillation frequency and amplitude of the liquid particle. At the same oscillation frequency, the energy transferred to the liquid increases significantly with the amplitude, leading to more cavitation.

### 4.2 Effects of the oscillation frequency on cavitation generation

To analyse the impact of the scaler's frequency on cavitation generation, we simulate the dental scaler oscillating in the periodontal pocket with both soft and nearly rigid boundaries at various frequencies *f* ranging from 20 to 45 kHz, with an amplitude of 0.01 mm.

**Fig. 10:**
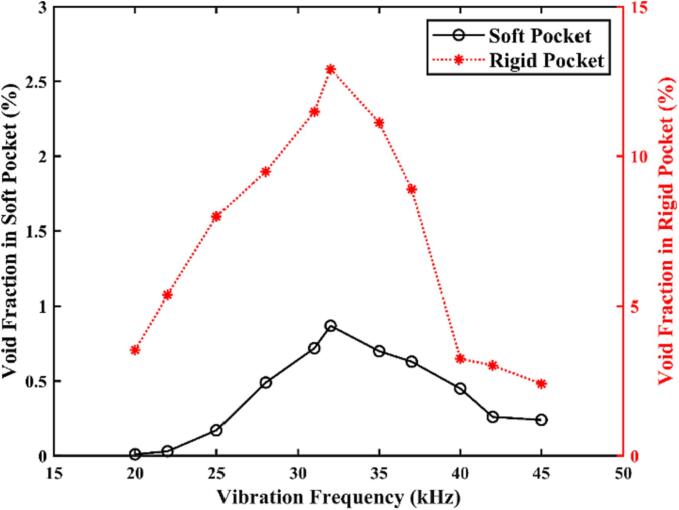
Void fraction in the periodontal pocket with both soft and nearly rigid boundaries plotted against the vibration frequency *f* of the scaler. The vibration frequency *f* ranges from 20 to 45 kHz, and other parameters are the same as in Fig. 5.

**Fig. 11:**
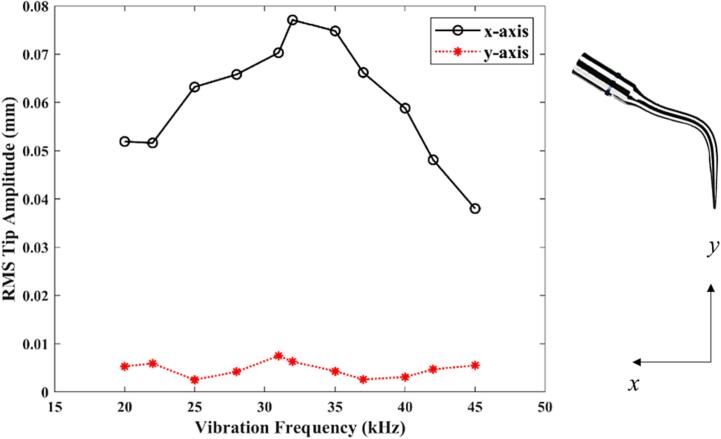
The root mean square (RMS) amplitude of the scaler tip along the *x*- and *y*- axes plotted against the vibration frequency *f* of the scaler tip. The vibration frequency *f* ranges from 20 to 45 kHz, and other parameters are the same as in Fig. 5.

As shown in Fig. 8, the void fraction within the periodontal pocket increases with the vibration frequency, attaining its peak value at approximately 32 kHz before gradually decreasing. Fig. 9 displays the root mean square (RMS) amplitude of the scaler tip along the *x-* and *y-* axes, which is evaluated by

(9) $$A_{\mathrm{RMS}}=\sqrt{\frac{1}{N}\sum_{i=1}^{N}A_{i}^{2}},$$

where *A_RMS_* represents the RMS amplitude, *A_i_* denotes the *i^th^* simulated amplitude, and *N* is the total number of samples. The amplitude along the *x*-axis follows the same trend as the void fraction and is significantly larger than that along the *y*-axis. To analyse this pattern, the natural frequency of the scaler undergoes vibrations without the presence of surrounding liquid is evaluated using ABAQUS which is 34.7 kHz. The natural frequency of the scaler undergoes vibrations in the liquid should decrease due to the effect of the added mass to the system by the liquid. Therefore, the peak value of the void fraction and scaler tip amplitude at 32 kHz in Fig. 10, Fig. 11 correspond to the resonance of the system subject to the oscillation of the scaler.

### 4.3 Effects of the cross-sectional shape of the scaler tip on cavitation generation

**Fig. 12:**
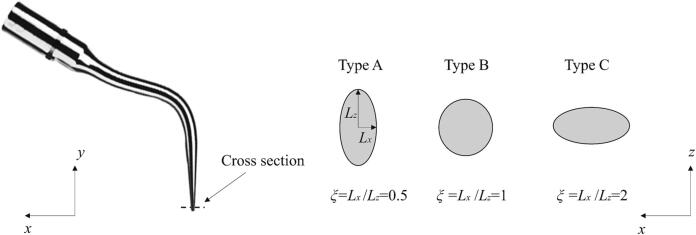
Schematics showing various cross-sectional shapes of dental scalers, where the *x*-axis is along the scaler tip displacement direction. Type A: a thin cross-section (*ξ* = 0.5), Type B: a circular cross-section (*ξ* = 1), and Type C: a blunt cross-section (*ξ* = 2).

We now analyse the impact of the cross-sectional shape of the scaler tip on cavitation generation. Three cross-sectional shapes are considered, as shown in Fig. 12, Type A: a thin cross-section to the direction of the displacement of the scaler tip, Type B: a circular cross-section, and Type C: a blunt cross-section. These three cross-sectional shapes are kept at the same area. The amplitude *A* and the frequency *f* are 0.01 mm and 31 kHz, respectively.

**Fig. 13:**
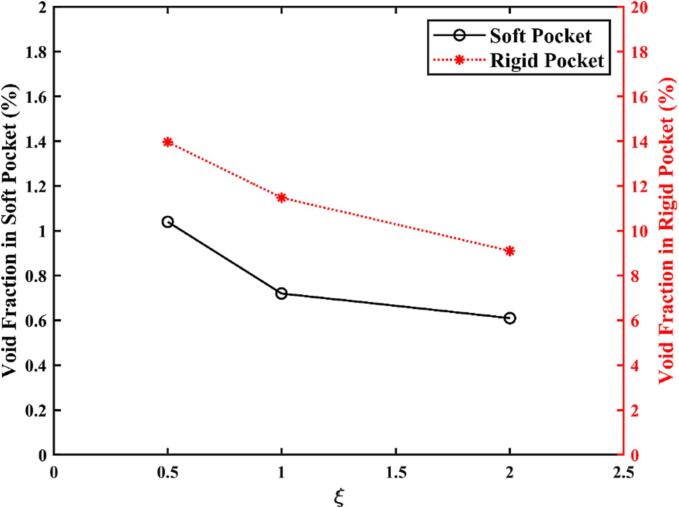
Void fraction in the periodontal pocket with both soft and nearly rigid boundaries, generated by dental scalers with different cross-sectional shapes (Type A: a thin cross-section *ξ* = 0.5, Type B: a circular cross-section *ξ* = 1, and Type C: a blunt cross-section *ξ* = 2). Parameters are the same as in Fig. 5.

**Fig. 14:**
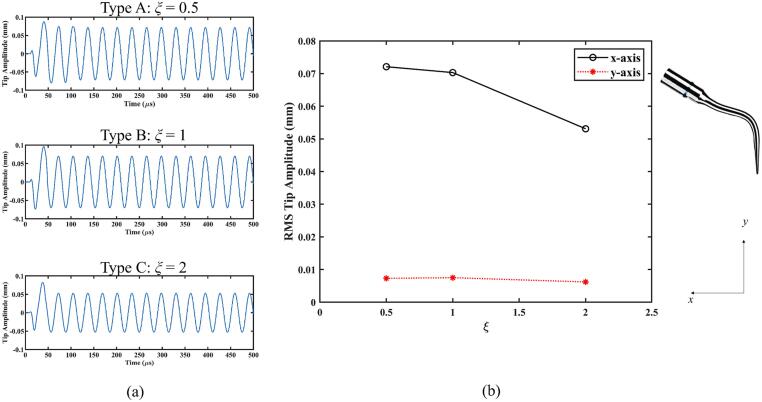
(a) The oscillation amplitude of the scaler tip along the *x*-axis with different cross-sectional shapes (Type A: a thin cross-section *ξ* = 0.5, Type B: a circular cross-section *ξ* = 1, and Type C: a blunt cross-section *ξ* = 2). (b) The RMS amplitude of the scaler tip along the *x-* and *y-* axes. Parameters are the same as in Fig. 5.

Fig. 13 illustrates that the void fraction in the periodontal pocket increases as the ratio *ξ* = *L_x_*/*L_z_* decreases. Fig. 14a illustrates the oscillation amplitude of the scaler tip along the *x*-axis for the three types of cross-sectional shapes, while Fig. 14b presents the RMS amplitude of the scaler tip along both the *x*- and *y*-axes. These amplitudes exhibit a similar trend to that of the void fraction. The moment of inertia of area (also known as the second moment of area) for the cross section of the scaler tip can be calculated by [38]

(10) $$I_{\mathrm{zz}}=\frac{\pi L_{x}^{3}L_{z}}{4}.$$

Following equation (10), the moments of inertia of area for Type A, Type B, and Type C are $I_{\mathrm{Azz}}=\frac{\pi R^{4}}{8}$, $I_{\mathrm{Bzz}}=\frac{\pi R^{4}}{4}$, and $I_{\mathrm{Czz}}=\frac{\pi R^{4}}{2}$, respectively, where *R* is the radius of the cross-section of Type B. According to the Euler–Bernoulli bending theory [39], the deflection *w* of a slender beam can be described by

(11) $$\frac{\partial^{2}w}{\partial y^{2}}=\frac{M}{\mathrm{EI}},$$

where *M* is the applied bending moment at given position, *E* is the Young's modulus of the material, and *y* is the position along the beam. The deflection of a bending beam is inversely proportional to the moment of inertia of area of the beam's cross-section. Therefore, with the same input power for the scaler, the scaler tip vibrates at a larger amplitude as the thickness along the main direction of oscillation (*x*-axis) decreases, leading to an increase in cavitation generation.

Manufacturers have designed different shaped tips for various cleaning applications inside the mouth. For instance, wider and larger tips are used to clean mineralized plaque deposits above the gum line, while much thinner tips are used for subgingival cleaning. To enhance the effective use of ultrasonic scalers in a non-touch mode, modifying the shape of the scaler tip to decrease the moment of inertia of area for the cross-section and increase the tip oscillation amplitude, resulting in more cavitation generation, can be beneficial.

## 5 Conclusions

Numerical simulations have been conducted to investigate cavitation generation within a periodontal pocket surrounded by soft gum tissue, induced by the oscillation of an ultrasonic dental scaler. Parametric studies have been undertaken to explore the impacts of vibration amplitude, frequency, and the cross-sectional shape of the dental scaler. The following new features/phenomenon are observed.

Cavitation generated in the periodontal pocket with a soft boundary is significantly less than that with a nearly rigid boundary. This is because a significant part of the acoustic energy is transformed into the elastic wave energy of the soft boundary.

At a fixed oscillation frequency, increasing the oscillation amplitude of the scaler leads to higher cavitation levels, as more ultrasonic energy is being introduced into the liquid.

Cavitation within the periodontal pocket reaches its peak when the vibration frequency is set at 32 kHz for the dental scaler tip 10P. This frequency closely matches the natural frequency of the scaler, and the peak cavitation coincides with the maximum amplitude of the scaler tip. This phenomenon is attributed to the resonance of the system.

The cross-sectional shape of the scaler tip significantly influences the oscillation amplitude of a scaler tip and the cavitation generation within the periodontal pocket. Keeping the cross-sectional area constant, an increase of the scaler tip's amplitude and cavitation generation can be achieved by reducing the thickness of the scaler tip along its main oscillation direction. This finding has the potential to optimize the structural design of dental scalers for better cleaning effects.

## Declaration of Competing Interest

The authors declare that they have no known competing financial interests or personal relationships that could have appeared to influence the work reported in this paper.
